# The known unknowns of the Hsp90 chaperone

**DOI:** 10.7554/eLife.102666

**Published:** 2024-12-31

**Authors:** Laura-Marie Silbermann, Benjamin Vermeer, Sonja Schmid, Katarzyna Tych

**Affiliations:** 1 Groningen Biomolecular Sciences and Biotechnology Institute, University of Groningen Groningen Netherlands; 2 Laboratory of Biophysics, Wageningen University & Research Wageningen Netherlands; University of Iowa United States; Goethe University Frankfurt Germany

**Keywords:** Hsp90, molecular chaperones, proteostasis

## Abstract

Molecular chaperones are vital proteins that maintain protein homeostasis by assisting in protein folding, activation, degradation, and stress protection. Among them, heat-shock protein 90 (Hsp90) stands out as an essential proteostasis hub in eukaryotes, chaperoning hundreds of ‘clients’ (substrates). After decades of research, several ‘known unknowns’ about the molecular function of Hsp90 remain unanswered, hampering rational drug design for the treatment of cancers, neurodegenerative, and other diseases. We highlight three fundamental open questions, reviewing the current state of the field for each, and discuss new opportunities, including single-molecule technologies, to answer the known unknowns of the Hsp90 chaperone.

Proteins are the active workforce in the cell. Among them, chaperone proteins are responsible for maintaining *proteostasis*, i.e., sustaining a functional proteome adapted to ever-changing cellular and environmental conditions. In eukaryotic cells, the heat shock protein 90 (Hsp90) plays a central role in proteostasis, as it provides a scaffold that binds substrate proteins (termed clients) as well as a variety of helper chaperones (known as cochaperones) (Picard, 2022; Noddings et al., 2021; Wang et al., 2021; Verba et al., 2016; Lee et al., 2021; Wen et al., 2023; Oberoi et al., 2022; García-Alonso et al., 2022). Hsp90 can therefore be regarded as a versatile workbench (Oberoi et al., 2022) where cochaperones act as tools to customise the chaperone’s function as needed for client recruitment (Wegele et al., 2006; Biebl et al., 2022; Taipale et al., 2012), (re)folding (Pirkl and Buchner, 2001; Riggs et al., 2007), (de)activation (Broemer et al., 2004; Belova et al., 2008), degradation (Dickey et al., 2007), or protection from degradation (Oberoi et al., 2022). Due to the many clients relying on its function, Hsp90 constitutes a proteostatic hub in eukaryotes (Taipale et al., 2012; Biebl et al., 2020), while only a handful of prokaryotic Hsp90 clients are known (Wickner et al., 2021). Among the hundreds of eukaryotic clients are transcription factors, signalling kinases, DNA replication proteins, cell division regulators, synapse proteins, and many more (Picard, 2022). Hsp90 is highly conserved throughout prokaryotes to higher eukaryotes (Chen et al., 2006), and appears to have back-transferred from eukaryotes to their archaeal precursors through horizontal gene transfer (Rebeaud et al., 2021). In the absence of stress, Hsp90 constitutes 1–2% of the protein mass in eukaryotic cells (Lai et al., 1984) and exists in super-stoichiometric ratios (∼2:1) to its cochaperones while it is outcompeted tenfold in concentration by that of all of its clients (Finka and Goloubinoff, 2013). Under stress conditions, Hsp90 expression is strongly upregulated (Borkovich et al., 1989). The active Hsp90 dimer (Wayne and Bolon, 2007) proceeds through an intricate functional cycle involving ATP hydrolysis, multiple conformational rearrangements, and (transient) protein-protein interactions, whose functional roles have remained largely enigmatic despite over 40 years of Hsp90 research (Ritossa, 1962; Finkelstein and Strausberg, 1983). This is surprising for an ATPase as prevalent as Hsp90, and clearly sets Hsp90 apart from many other important ATPases with very well-understood molecular mechanisms. In this Review, we highlight the most important ‘*known unknowns*’ of Hsp90’s molecular function, with the goal of inspiring new routes to address them and advance the field (Figure 1).

**Figure 1.**
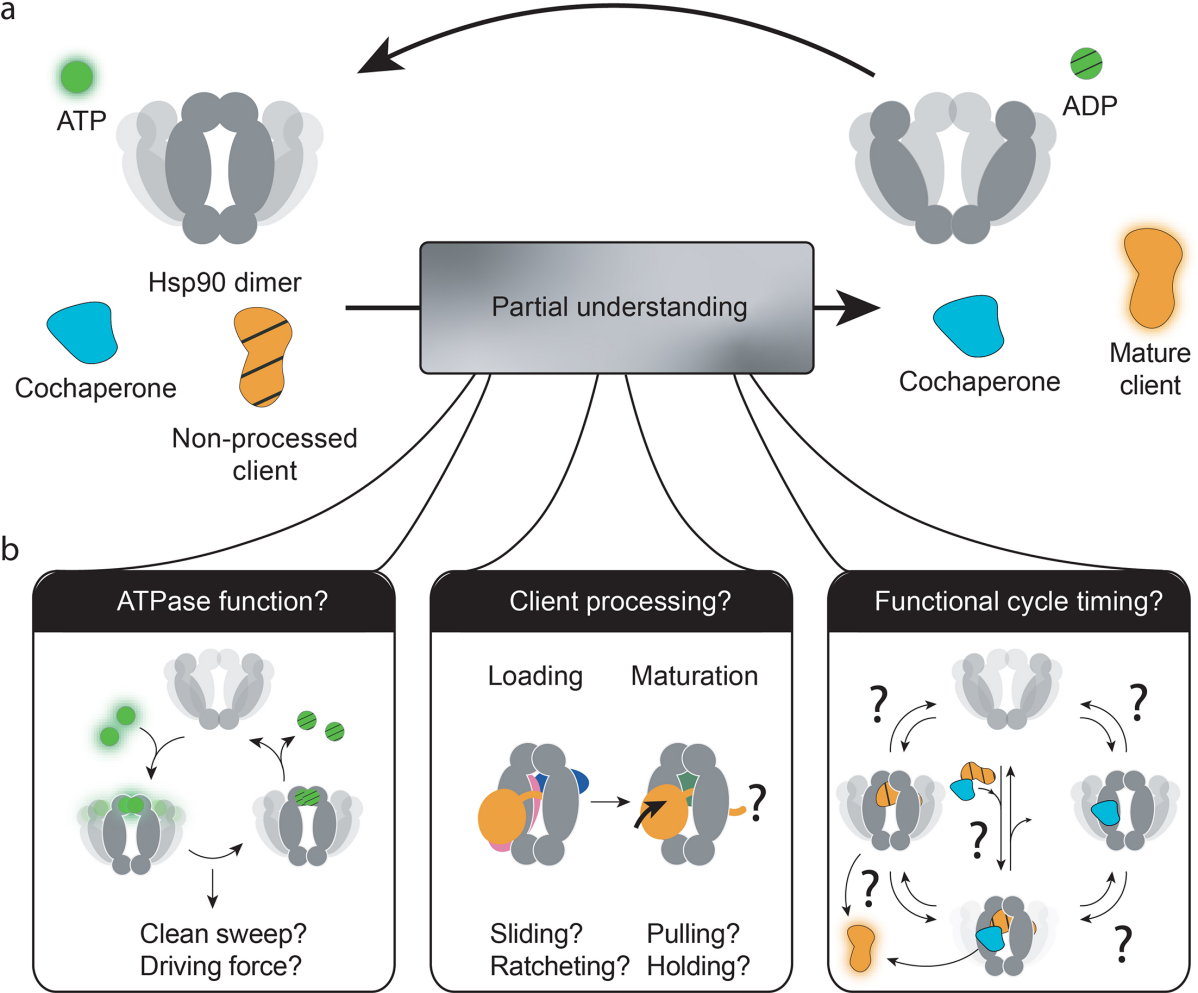
Three central questions about heat-shock protein 90’s (Hsp90’s) functional mechanism. (**a**) The current combined knowledge describes a general functional cycle of eukaryotic Hsp90, where Hsp90 hydrolyses ATP at a slow rate, interacts with cochaperones and clients, and (by only partially understood mechanisms) matures clients to their functional form. (**b**) Several open questions remain, as illustrated: What is the role of Hsp90’s ATPase activity? How does Hsp90, alone or in cooperation with cochaperones, process its vast client base? Which is the sequence of interactions by which the Hsp90 system operates to produce functional clients? And which are reversible processes as compared to unidirectionally driven ones? Which are fast or rate-limiting steps?.

Structurally, Hsp90 monomers consist of three domains (Figure 2A): an N-terminal domain (NTD), a middle domain (MD), and a carboxy-terminal domain (CTD), as well as an unstructured charged linker region between the NTD and MD. The NTD contains an ATP-binding pocket and a flexible loop segment, the so-called lid, which can fold over the ATP pocket (Prodromou et al., 2000; Schulze et al., 2016; Henot et al., 2022). The charged linker modulates Hsp90 function via NTD interactions (Hainzl et al., 2009; Jahn et al., 2014) and can influence client binding (López et al., 2021). Most known client binding sites are located in the MD (Noddings et al., 2021; Wang et al., 2021; Verba et al., 2016; Oberoi et al., 2022; García-Alonso et al., 2022; Lorenz et al., 2014; Radli and Rüdiger, 2018), while specific MD residues also participate in nucleotide binding, alongside NTD residues (Mader et al., 2020). The CTD provides the main dimerisation interface (Minami et al., 1994), and can participate in client interactions (Verba et al., 2016; Lorenz et al., 2014). In cytosolic eukaryotic Hsp90, the CTD contains an additional recognition motif (MEEVD) (Biebl and Buchner, 2019) for tetratricopeptide repeat-containing cochaperones, such as Sti1/Hop and Cpr6 (Schopf et al., 2017). The Hsp90 dimer undergoes large conformational rearrangements, involving conformational ensembles with several open (V-shaped), intermediate, and compacted dimer structures (Schopf et al., 2017; Ali et al., 2006; Shiau et al., 2006; Mickler et al., 2009; Ratzke et al., 2012b), as illustrated in Figure 1 (grey shading). Hsp90 is also assisted by cochaperones that either directly modify clients, or interact with Hsp90 to modulate client processing (Röhl et al., 2013), resulting in a vast variety of transiently occurring chaperone complexes. Post-translational modifications (PTMs) provide an additional level of regulation of Hsp90 function and we refer the reader to a recent comprehensive review of PTMs on Hsp90 (Backe et al., 2020). For example, specific PTMs alter Hsp90’s ATPase activity (Backe et al., 2023), exert allosteric effects (Rehn et al., 2016; Zuehlke et al., 2017), may confer client specificity to Hps90 (Scroggins et al., 2007), target Hsp90 for degradation (Mollapour et al., 2010), and regulate its interaction with clients (Backe et al., 2023; Zuehlke et al., 2017; Scroggins et al., 2007; Rani et al., 2024; Xu et al., 2012; Duval et al., 2007; Burmann et al., 2020), cochaperones (Mollapour et al., 2010; Xu et al., 2012; Vaughan et al., 2008; Bachman et al., 2018; Mollapour et al., 2014), and small-molecule inhibitors (Mollapour et al., 2014; Beebe et al., 2013). Further increasing the complexity is asymmetry in the Hsp90 dimer and its interactions (Mayer and Le Breton, 2015). Examples include the individual ATP hydrolysis by Hsp90 monomers within a dimer (Mishra and Bolon, 2014; Huang et al., 2019; D’Annessa et al., 2021; Wolf et al., 2021; Elnatan et al., 2017), asymmetric binding of cochaperones to the Hsp90 dimer (Noddings et al., 2021; Wang et al., 2021; Verba et al., 2016; Lee et al., 2021; Wen et al., 2023; Oberoi et al., 2022; García-Alonso et al., 2022; Retzlaff et al., 2010), asymmetric PTMs of the Hsp90 dimer (Mollapour et al., 2014), asymmetric client binding (Finci et al., 2024), and the increase of Hsp90 dimer ATPase activity through asymmetry in N-terminal β-strap dynamics (Magnan et al., 2024). Given this polymorphic behaviour, it has been impossible to define a single functional cycle for Hsp90, which – alongside other peculiarities discussed below – limits the current understanding of the Hsp90 functional mechanism.

**Figure 2.**
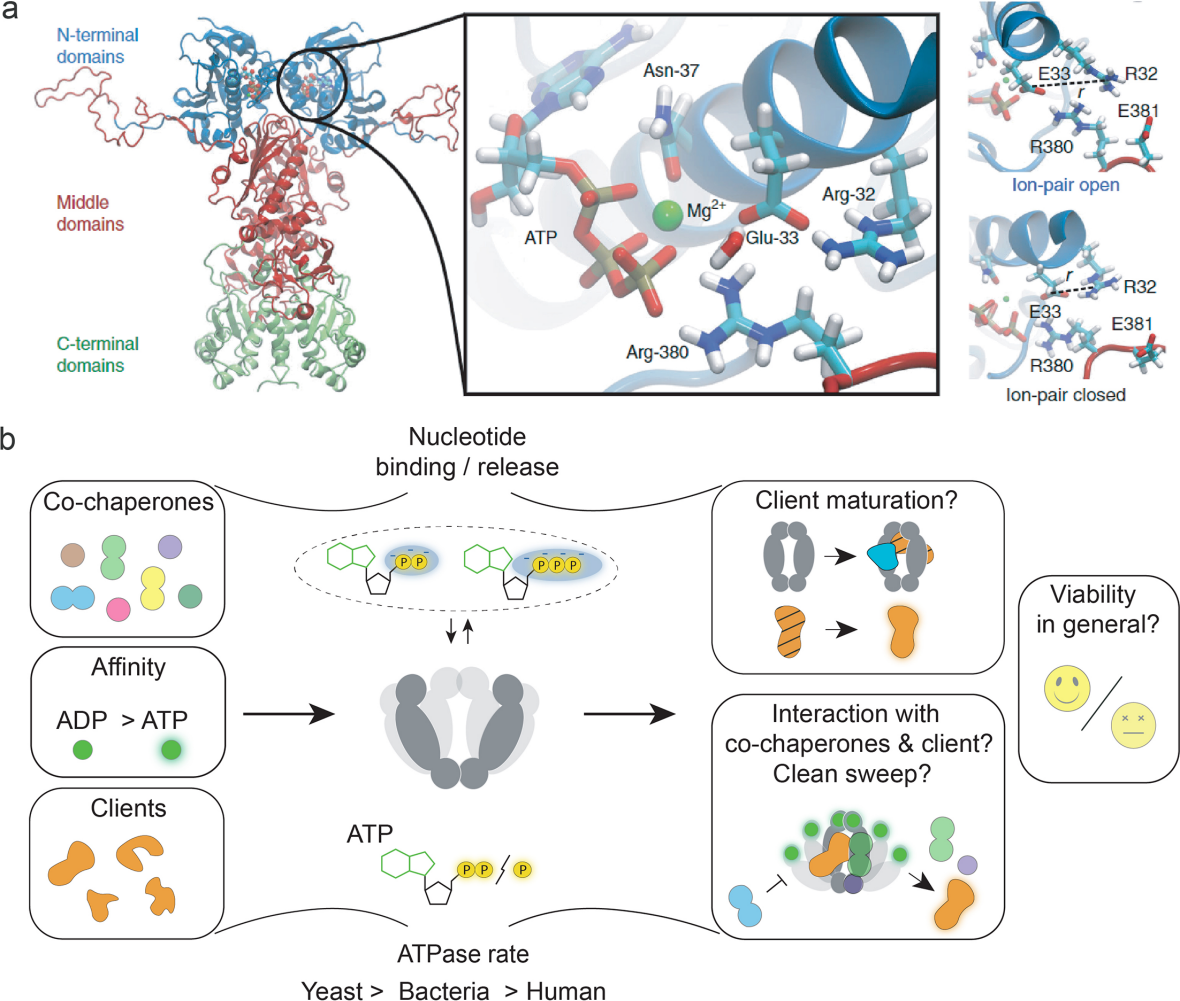
The role of heat-shock protein 90’s (Hsp90’s) ATPase function. (**a**) Structure of the yeast Hsp90 dimer (PDB ID: 2CG9, Ali et al., 2006). The insert obtained from an molecular dynamics (MD) simulation shows the active site with bound ATP. MD simulations indicate a role of ion pair opening in the ATP hydrolysis reaction. Panel A is reproduced from Figures 1 and 3 from Mader et al., 2020. (**b**) Visual summary of Hsp90’s ATPase rate modulation by interactors, ATPase rate differences among different species, and partially understood aspects of Hsp90’s ATPase function, such as interactor dissociation. The notion that ATP hydrolysis is essential for yeast cell viability was challenged by recent evidence. Nucleotide binding and release may be sufficient to support yeast cell viability.

## Peculiarities of the Hsp90 protein system

For common substrate-binding ATPase proteins, such as proteases, helicases, motor proteins, etc., the ATPase mechanism and function are well defined. In stark contrast, Hsp90 performs various functions and (likely for that reason) is less well understood. A comparative overview of Hsp90 and a selection of well-known ATPases (Table 1) reveals several unique features of Hsp90.

**Table 1.** Special features of heat-shock protein 90 (Hsp90) compared to other well-known ATPase proteins (model ATPases): (i) the motor protein kinesin, (ii) the helicase DnaB, (iii) the protease ClpXP.

Functional feature	Eukaryotic Hsp90	Model ATPases
Mechanistic role of ATPase:	Only partially understood (Kirschke et al., 2014; Zierer et al., 2016; Reidy et al., 2023)	Understood hand-over-hand transport (Yildiz et al., 2004; Hancock and Howard, 1999) DNA unwinding (Biswas and Biswas, 1999) unfolded protein translocation (Cordova et al., 2014)
ATPase rate*:	Slow: 0.1–5/min (Lorenz et al., 2014; Falsone et al., 2009; Lopez et al., 2021; Street et al., 2011)	Fast: 1–100/s (Hunter and Allingham, 2020; Hackney, 1988; Aubin-Tam et al., 2011; Henderson et al., 2011; Rajendran et al., 2000)
Substrate specificity:	Binds diverse clients (Picard, 2022)	Specific binding of microtubules (Hancock and Howard, 1999) DNA (Biswas and Biswas, 1999), or recognition motifs (Chan et al., 2014)
Interaction affinities:	Low (Lorenz et al., 2014; Karagöz et al., 2014)	Medium to high (Woehlke et al., 1997; Biswas and Biswas-Fiss, 2006; Singh et al., 2000; Baker and Sauer, 2012)
ATPase functional cycle:	Directionality not well defined (Ratzke et al., 2012a; Zierer et al., 2016)	Strict directional order (Cordova et al., 2014; Hunter and Allingham, 2020; Wiegand et al., 2019)

* ATPase in the presence of substrate.

First, Hsp90’s ATP hydrolysis rate is very slow (0.1–1.5/min) (Wayne and Bolon, 2007; Panaretou et al., 1998; Siligardi et al., 2002; Richter et al., 2001) – tens to hundreds of times slower than other ATPases in both the presence or absence of substrate. This is a common feature of the so-called GHKL family (including the eponymous DNA *g*yrase, *H*sp90, histidine *k*inase (HisK), and Mut*L*), which share slow ATPase rates [generally 0.02–10/min (Surette et al., 1996; Childers et al., 2014; Yang et al., 2022), and DNA gyrase with ~100/min (Maxwell and Gellert, 1984)]. Second, the role of ATP hydrolysis is well understood in the molecular mechanism of many standard ATPases. Contrastingly, the function of ATP hydrolysis by Hsp90 has remained unclear, and several hypotheses have been posed by the Hsp90 field, which we discuss below.

Next, the *molecular mechanisms* by which Hsp90 chaperones its clients are not well understood, since often only the *outcomes* (e.g. a functional client) are measurable, but not the molecular process leading there. This contrasts with most ATPases, where the molecular mechanism is well defined (e.g. myosin’s power stroke [Piazzesi et al., 2002], kinesin’s hand-over-hand cargo transport [Yildiz et al., 2004], or protein threading through protease rings for degradation [Cordova et al., 2014]). Moreover, most ATPases show a specific reaction coordinate (e.g. directional translocation of an unfolded protein [Cordova et al., 2014], or sliding along DNA [Gradia et al., 1999]). For Hsp90, no single reaction coordinate seems to exist, given that it interacts with a broad clientele to perform varied chaperone functions with distinct (client-specific) sets of cochaperones involved (Picard, 2022). This is very different from standard ATPases recognising one specific substrate or motif. These numerous interactions occur with low affinity up to the micromolar range (Lorenz et al., 2014; Biebl and Buchner, 2019; Karagöz et al., 2014) and across the entire protein, not just at one specific client-binding site. While some clients bind within the cleft between both monomers of N-terminally (semi-)closed Hsp90 referred to as the lumen (Noddings et al., 2021; Wang et al., 2021; Verba et al., 2016; Oberoi et al., 2022; Prodromou et al., 1997a) others were found bound to N-terminally open Hsp90 (Karagöz et al., 2014; Oroz et al., 2017). Cochaperones interact with all domains of Hsp90. Ultimately, Hsp90 achieves the seemingly impossible, namely, to chaperone structurally and functionally diverse clients in *client-specific* ways (Karagöz et al., 2014; Verba and Agard, 2017), which is assumed to be orchestrated via its many cochaperone interactions.

Lastly, for many proteins the timing of their functional cycle is well understood: which functional states are involved (including rare, potentially rate-limiting intermediates), at what rates they occur, whether they occur sequentially or reversibly, and which of them are deterministically driven by ATP hydrolysis (Table 1, ATPase mechanistic role and functional cycle). This quantitative information provides a mechanistic understanding of the molecular system which aids rational drug and therapy design. As illustrated in Figure 1, for Hsp90, this detailed molecular level of understanding is yet to be achieved, which may have had an impact on the relatively low success of Hsp90-targeted drugs up to now (Xiao and Liu, 2020; Chiosis et al., 2023). However, a vast body of existing biochemical and structural results about client-specific functional cycles exists and indicates possible routes forward.

## The enigmatic role of Hsp90’s ATPase function

The precise functional role of the slow ATPase of Hsp90 has remained unsolved despite intense research. We first discuss the structural basis of ATP binding and the hydrolysis mechanism. Hsp90 binds ATP in a unique Bergerat fold (Dutta and Inouye, 2000). The active site of ATP hydrolysis in yeast Hsp90 comprises five amino acid residues (Figure 2a; Mader et al., 2020): in the NTD R32, E33, and N37, and in the MD R380 (Schulze et al., 2016; Ali et al., 2006) and E381 (Mader et al., 2020). N37 is involved in coordinating ATP via a magnesium ion (Prodromou et al., 1997b). Hybrid quantum/classical (QM/MM) free-energy calculations combined with large-scale atomistic molecular dynamics simulations (Mader et al., 2020) suggest that, upon opening of the R32-E33 ion pair (Figure 2a, rightmost panel) via long-range interaction and conformational switching, E33 deprotonates a water molecule in the active site, resulting in a hydroxide ion which is stabilised by R380, subsequently allowing nucleophilic attack of the bond linking the β- and γ-phosphate in the bound ATP, in which the magnesium ion coordinated by N37 pulls away electron density from the γ-phosphate to lower the hydrolysis energy barrier. After hydrolysis, R380 stabilises the resultant cleaved inorganic phosphate (Mader et al., 2020). The coordinated structural changes required for ATP hydrolysis are understood to be rate-limiting for Hsp90’s slow ATPase cycle (Schulze et al., 2016; Schubert et al., 2021; Richter et al., 2008; Hessling et al., 2009). In other words, the probability of being in the hydrolysis competent state (Schmid and Hugel, 2020) is low due to the general flexibility of Hsp90 and, in particular, its split ATP-binding site (Ali et al., 2006) composed of residues of both the NTD and MD. Several single-molecule studies provide deeper insight into the flexibility of Hsp90 on various timescales and in relation to its ATPase activity (Schulze et al., 2016; Mickler et al., 2009; Ratzke et al., 2012b; Huang et al., 2019; Schubert et al., 2021; Ratzke et al., 2014; Ratzke et al., 2012a; Vollmar et al., 2024; Tych et al., 2018), as detailed in the section on single-molecule observations. Last but not least, the measured ca. threefold higher affinity for ADP than ATP (Prodromou et al., 1997a; Weikl et al., 2000) implies a considerable product-inhibited fraction of ADP-bound Hsp90 even in the presence of excess ATP, which is expected to affect interactor binding and client maturation also in vivo (Mader et al., 2020).

A number of functional roles of Hsp90’s ATPase activity has been discussed. For example, it was suggested that it facilitates the dissociation of interaction partners during GR maturation (Kirschke et al., 2014) (illustrated in Figure 2b). Interestingly, also for MutL – another member of the GHKL class with a similar ATP-binding site and hydrolysis rate – ATPase activity induces (DNA) substrate dissociation (Yang et al., 2022). In the Hsp90-Cdc37-kinase cycle, Cdc37 inhibits Hsp90’s ATPase (Siligardi et al., 2002; Roe et al., 2004) while clients are held in the complex (Verba and Agard, 2017). Similarly, p23 inhibits the Hsp90 ATPase during GR processing (McLaughlin et al., 2002; Richter et al., 2004). This suggests a role of ATP hydrolysis in regulated client dissociation, which is further supported by molecular dynamics simulations (D’Annessa et al., 2021). Going further into molecular detail, ATP hydrolysis by only one of the Hsp90 monomers was suggested to induce a conformational change that collapses a client binding site in the lumen of Hsp90 as found by single-molecule Förster resonance energy transfer (smFRET) combined with molecular dynamics simulations (Wolf et al., 2021). While for some clients, ATP hydrolysis by Hsp90 was found to be necessary (e.g. folding of luciferase [Morán Luengo et al., 2018] and reversal of Hsp70-induced inhibition of GR activation by Hsp90 [Kirschke et al., 2014]), in other cases, only ATP binding but not hydrolysis is required for client processing (e.g. cochaperone-independent p53 processing [Walerych et al., 2010] and stabilisation of the client kinase v-Src at elevated temperatures [Boczek et al., 2015]). Notably, ATP binding was found to reduce luciferase binding affinity in Grp94, suggesting bound ATP may be important in promoting chaperone-mediated client folding (Amankwah et al., 2024). In addition, Hsp90 and its clients *mutually* affect each other and some clients alter Hsp90’s ATPase rate: interestingly, ATPase stimulation (α-synuclein [Falsone et al., 2009] and ribosomal protein L2 [Motojima-Miyazaki et al., 2010]) as well as reduction (GR-LBD client [Lorenz et al., 2014; Lopez et al., 2021]) have been observed. Both effects were attributed to conformational stabilisation of either ATPase competent or non-competent states, respectively. In this way, some clients have been suggested to modulate their own residence time on Hsp90 (Lorenz et al., 2014). Other clients, such as the mineralocorticoid receptor (MR)-LBD, Tau, and the p53-DNA-binding domain (DBD) seem not to affect Hsp90_euk_’s ATPase activity (Lopez et al., 2021).

Interestingly, smFRET has revealed differences between prokaryotic and eukaryotic Hsp90 regarding the mechano-chemical coupling of ATP hydrolysis and large-scale structural rearrangements of the Hsp90 dimer (V-shaped opening/closing). Prokaryotic Hsp90 (subsequently referred to as HtpG) is described to function as a Brownian ratchet, where thermal fluctuations are biased by ATP-binding towards the closed state, providing a degree of directionality to its conformational cycle (Ratzke et al., 2012b). This has not been observed for eukaryotic Hsp90 (Hsp90_euk_) where thermal fluctuations dominate even in the presence of ATP (Ratzke et al., 2012a; Zierer et al., 2016), and its conformational equilibrium is more affected by cochaperone and client interactions, as well as molecular crowding (Lorenz et al., 2014; Schmid and Hugel, 2020; Ratzke et al., 2014; Lopez et al., 2021). In addition, smFRET showed that Hsp90 inhibitors (binding to either the Bergerat fold, or to the Hsp90 C-terminal domain) have little effect on Hsp90_euk_’s conformational dynamics (Schmid et al., 2018), although environmental conditions (e.g. the presence of point mutations, crowding agents, or cochaperones) do influence Hsp90’s conformational kinetics (Schmid and Hugel, 2020). Nevertheless, Hsp90_euk_’s nucleotide state modulates the cochaperone- and therefore client-interaction affinities. For example, ATP binding induces NTD rotation in Hsp90_euk_, affecting distal client binding sites through long-range allosteric communication, increasing the binding affinity of some (GR-LBD) but not all studied clients (p53-DBD, Tau) (Karagöz et al., 2014; Lopez et al., 2021).

Altogether, the large variation of reported effects of Hsp90’s ATPase activity remains puzzling. This is even more so, in light of the unsolved question: is Hsp90’s ATPase activity indeed essential for cell viability? Activator of Hsp90 ATPase activity 1 (Aha1) is the only known cochaperone that significantly accelerates Hsp90 ATPase activity (Panaretou et al., 2002). Modified expression levels of Aha1 have been shown to significantly affect Hsp90 client maturation (Shelton et al., 2017; Wang et al., 2006), suggesting that Hsp90 ATPase activity plays a notable role in this process. It has also been hypothesised that Aha1 influences client maturation by modulating the residence time of clients on Hsp90 (Wang et al., 2006). While, up to now, most reports maintain the notion that ATPase activity is essential for Hsp90’s function and viability of the eukaryotic cell (Panaretou et al., 1998; Obermann et al., 1998), recent evidence challenges this view: a hydrolysis-dead yeast Hsp90 E33A mutant (Zierer et al., 2016) as well as several Hsp90 orthologs with this mutation (Reidy et al., 2023) were found to still support yeast cell growth. The findings of these studies imply that the ability to adopt different conformations is enough for some functions of Hsp90, and that nucleotide association and dissociation may be sufficient for regulation of Hsp90’s conformational equilibrium, while hydrolysis may play a different role.

In summary, the large body of experimental evidence for (i) the mutual effects of cochaperones and clients on Hsp90’s ATPase rate, and for (ii) the role of ATP hydrolysis in client processing and cell viability, raise new questions and we suggest single-molecule experiments to address them in Box 1.

Box 1. **New opportunities to elucidate heat-shock protein 90’s (Hsp90’s) enigmas with single-molecule resolution**

1. The Hsp90 ATPase has been hypothesised to function as a release trigger for cochaperones and/or clients. Caged ATP allows for spatiotemporal control over ATP hydrolysis, and using single-molecule experiments (Sabantsev et al., 2022), it offers an assay for cochaperone or client release upon laser-triggered ATP hydrolysis by Hsp90.
2. Questions about Hsp90’s ATPase and how it is coupled to Hsp90’s conformational state can be elucidated by investigating the differences between prokaryotic and eukaryotic Hsp90, e.g., using chimeras. smFRET studies revealed that closing of *Escherichia coli* HtpG shows clear ATP dependence (Ratzke et al., 2012b), whereas yeast Hsp82 closing is only weakly dependent on ATP even in the excess presence of the nucleotide (Mickler et al., 2009). smFRET and force spectroscopy can directly observe such differences in conformational dynamics and reveal the energies involved.
3. Client processing by Hsp90 can be observed at the single-molecule level in real time, e.g., to probe the proposed sliding hypothesis for GR processing (Noddings et al., 2021). Such nanometer distance changes induced by sliding or ratcheting can be observed by smFRET within one chaperone complex. Force spectroscopy can also be used to detect distance changes associated with client remodelling.
4. Functional cycle timing, the order of individual events, and whether they occur directionally or reversibly can be observed by single-molecule fluorescence and dye-labelled interactors (e.g. Hsp90, client, Hsp70, p23, etc.) at physiological concentrations (using zero-mode waveguides [ZMW]). The impact of clients and cochaperones on the conformational state of Hsp90 can also be probed by using force spectroscopy experiments combined with microfluidics.

## Client processing by Hsp90

**Figure 3.**
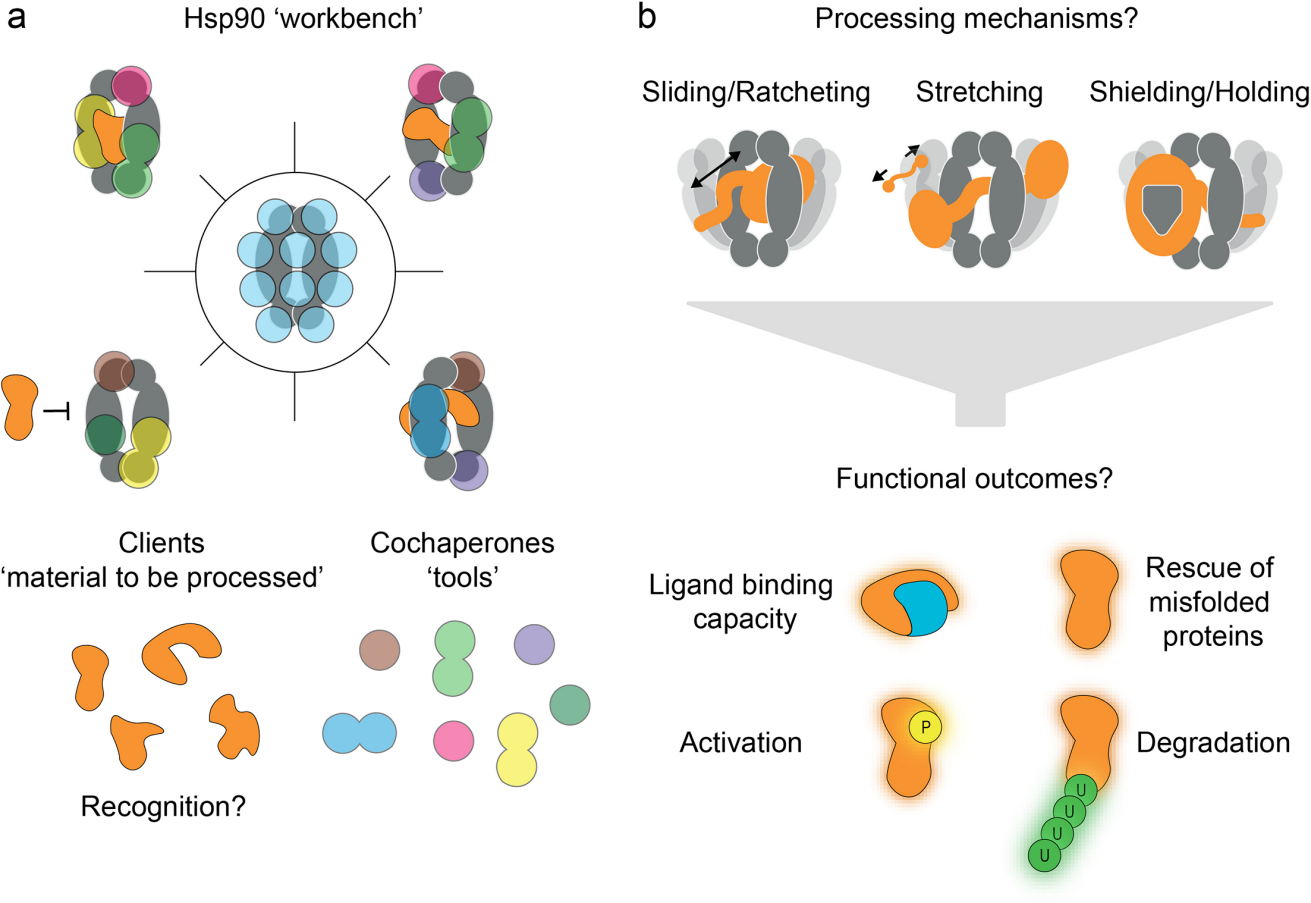
Convergent model of heat-shock protein 90’s (Hsp90’s) functional mechanism. (**a**) Hsp90 can be regarded as a workbench, bringing clients and cochaperones (coloured tools) into close proximity via interaction sites spread over the entire protein (blue spots). However, many details of recognition of the numerous clients by Hsp90 and its cochaperones remain elusive. (**b**) Various client processing modes are being discussed based on growing experimental evidence, such as sliding, ratcheting, stretching, shielding, and holding. These processing modes seem to be client-specific, and they may offer distinct functional outcomes (e.g. misfolding rescue, or targeting for or protection from degradation).

Client processing by Hsp90_euk_ can be classified into refolding (Kirschke et al., 2014; Morán Luengo et al., 2018), holding (Freeman and Morimoto, 1996), sorting for degradation (Dickey et al., 2007; Karagöz et al., 2014; Falsone et al., 2009), ligand binding regulation (Kirschke et al., 2014; Pratt and Dittmar, 1998; Ghosh and Stuehr, 2012), re-/de-activation (Broemer et al., 2004; Belova et al., 2008; Mu et al., 2023; Sato et al., 2000), and regulation of assemblies (Zhao et al., 2008; Manjarrez et al., 2014). Hsp90’s diverse clients differ both structurally and functionally (Taipale et al., 2012; Pearl and Prodromou, 2006; McClellan et al., 2007) and, related to that, Hsp90 does not have a well-defined client binding site, which is in contrast to other chaperones, such as Hsp70. Instead, Hsp90 clients form numerous low-affinity contacts through hydrophobic interactions (Babu and Freeman, 2024) and some client-specific charge-charge interactions (Radli and Rüdiger, 2018). Certain clients (e.g. GR and kinases) primarily bind to Hsp90’s lumen formed by both MDs, while intrinsically disordered proteins (IDPs, e.g. Tau, misfolded transthyretin, etc.) bind interfaces extending from the MD to the NTD (Oroz et al., 2017; Rutledge et al., 2022). Despite the breadth of clients, Hsp90 is understood to exhibit client-specific chaperoning action, presumably through specific sets of cochaperones (Noddings et al., 2021; Wang et al., 2021; Verba et al., 2016; Lee et al., 2021; Wen et al., 2023; Oberoi et al., 2022; Liu et al., 2020). Indeed, only certain cochaperones function as integral components of the Hsp90_euk_ system, while others are client-specific and provide Hsp90 with the necessary plasticity (Sahasrabudhe et al., 2017; Radli and Rüdiger, 2017; Figure 3a), e.g., through specific client recruitment (Taipale et al., 2012) or by modulating the time for which a client is bound to Hsp90 (Biebl et al., 2020; Wang et al., 2006; Sahasrabudhe et al., 2017; Prince and Neckers, 2011; Koulov et al., 2010).

Exciting molecular-level details of client processing have recently been revealed for the GR, Hop/Sti, p23 system in 3D structures of these multi-component complexes, solved by the Agard lab (Noddings et al., 2021; Wang et al., 2021) with cryo-EM. They represent two snapshots along the complex client processing trajectory, termed the loading complex (Wang et al., 2021) and the maturation complex (Noddings et al., 2021). Together, they suggest a sliding or ratcheting movement of the client GR through the Hsp90 lumen. Likewise, three kinase clients (BRAF_V600E_ [Oberoi et al., 2022], Cdk4 [Verba et al., 2016], RAF1 [García-Alonso et al., 2022; Finci et al., 2024]) and the aryl hydrocarbon receptor (Wen et al., 2023) also thread through the Hsp90 lumen. Such molecular clamping by Hsp90 was suggested to keep the domains of multidomain clients separated, thus facilitating their independent folding (Noddings et al., 2021) and preventing inter-domain misfolding (Figure 3b). Destabilised kinases are thought to be held by Hsp90 to facilitate their reactivation (Verba and Agard, 2017), after local kinase motions poise the kinase for Hsp90 processing (Keramisanou et al., 2022). During the processing of Tau, which binds to open Hsp90_euk_ in numerous conformations (Karagöz et al., 2014), Hsp90 has been hypothesised to act as a holdase, preventing the formation of toxic oligomeric species (Rutledge et al., 2022). However, the precise role of Hsp90 in Tau aggregation is still a topic of ongoing research, as discussed in a recent review (Ramirez and Zweckstetter, 2023). For example, it was also found that Hsp90 recruits CHIP resulting in the ubiquitination and degradation of Tau (Dickey et al., 2007). For the p53 DBD, smFRET measurements revealed considerable dynamics in the presence of all relevant (co-)chaperones, suggesting that p53 conformation is constantly remodelled by Hsp70/Hsp90 (Dahiya et al., 2019). Overall, Hsp90’s structural plasticity allows clients and cochaperones to individually orchestrate client-processing mechanisms, making it a flexible master chaperone.

The energetic driving forces for Hsp90’s diverse molecular modes of action – folding, holding, stretching, etc. – result from different underlying kinetics of inter- and intra-molecular interactions. For example, the balance between client holding versus folding depends on the (forward and reverse) kinetic rate constants of the two processes: if the client-folding step is slower than client (re-)binding to the chaperone, the holdase function is kinetically favoured and folding is rate-limiting for that particular client (Kawagoe et al., 2022). The sequential refolding of individual structural elements of the client can decrease their local affinity for Hsp90, enabling gradual dissociation from Hsp90. This process allows clients to sample a wide range of conformations prior to dissociating from Hsp90 (Qu et al., 2024). Transient interactions are prerequisite for this dynamic model. Thermodynamically, transient interactions can result in avidity effects where multiple low-affinity contacts add up, resulting in high affinities with down to nanomolar dissociation constants (K_d_) (Nooren and Thornton, 2003). While such strong avidity is characterised by large interfaces (∼1500 Å) (Noddings et al., 2023; Nooren and Thornton, 2003), the low affinities of Hsp90 to its clients (micromolar K_d_) (Lorenz et al., 2014; Karagöz et al., 2014) likely result from much smaller interfaces.

For folding processes to occur, specific energy barriers need to be overcome. While small conformational changes have low energy barriers and occur spontaneously in thermal equilibrium, large rearrangements with higher energy barriers are not accessible by thermal energy alone (1 k_B_T≈ 4.1×10^−21^ J **≙** 0.6 kcal/mol). In other words, although folding is an exothermic process (cf. Anfinsen’s dogma [Anfinsen, 1973]), energy barriers can kinetically trap un- or misfolded states and external energy is often required for efficient folding. This can be binding energy from molecular interactions (including cochaperone binding), ATP hydrolysis energy, or potential energy changes provided by covalent PTMs. Notably, for its client-specific function, Hsp90 exploits all of these modes of action in one unified system, and first efforts have been made to directly quantify the energies involved (Mashaghi et al., 2022). Overall, Hsp90_euk_ has emerged as a highly multi-functional workbench that uses cochaperones as tools for the customised processing of its diverse clients. Many proposed models for client processing are yet to be experimentally proven. New routes to address this using single-molecule approaches are discussed below and in Box 1.

## The timing of Hsp90’s functional cycle

**Figure 4.**
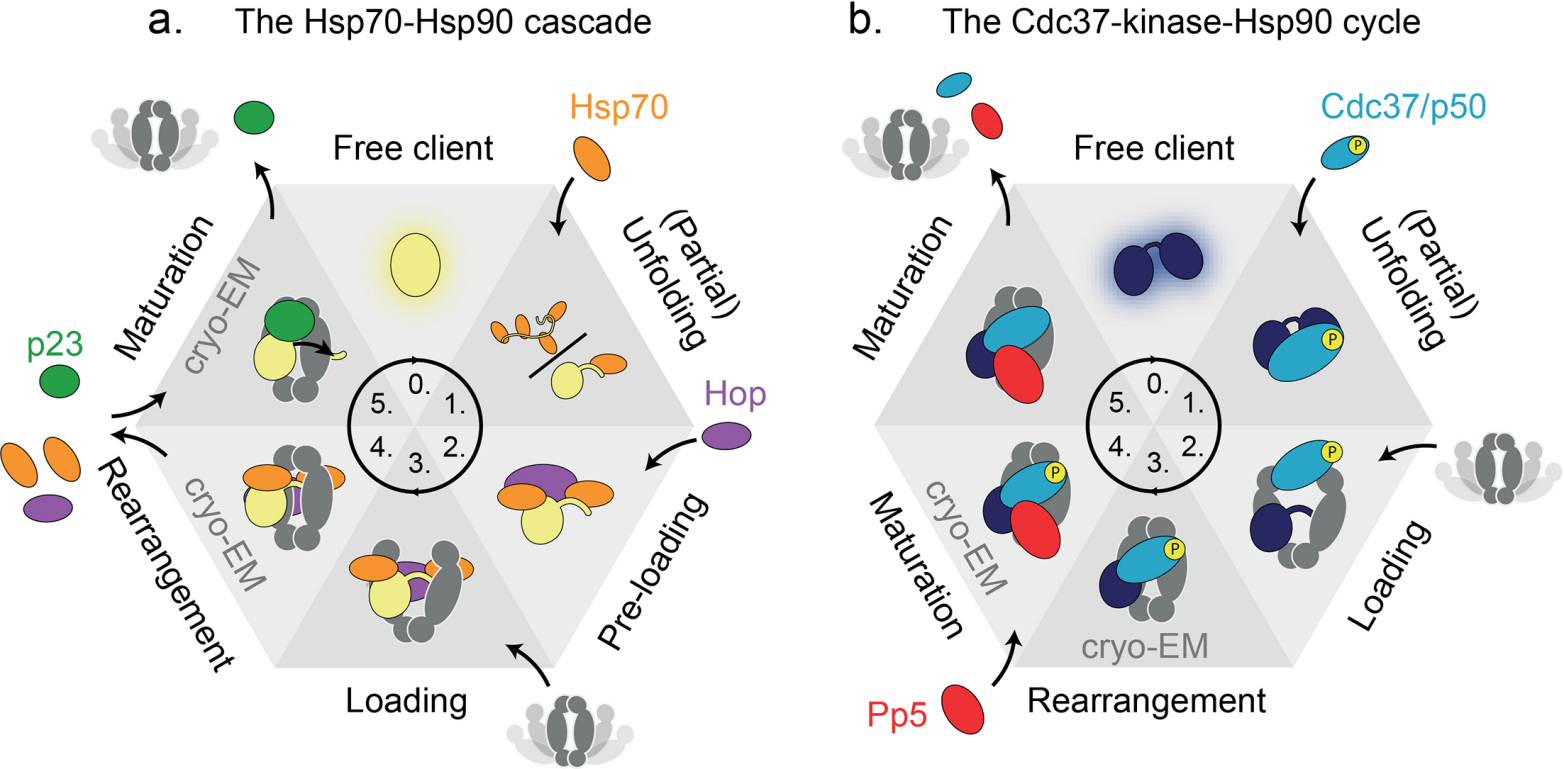
Heat-shock protein 90 (Hsp90’s) functional cycle timing illustrated by two literature examples. Schematic representation of the two discussed functional cycles of eukaryotic Hsp90, illustrated using six states each. Cryo-EM structures of two of the states exist, as indicated. Many transient interactions and intermediate complexes limit the current understanding of the order and timing of events, as well as their directionality or reversibility. (**a**) The Hsp70-Hsp90 cascade and (**b**) the Cdc37-kinase-Hsp90 cycle. Both cycles have in common that the client is (partially) unfolded prior to recruitment to Hsp90 in an intermediate loading complex. Next, by unknown mechanisms, clients are processed by Hsp90 through at least one intermediate maturation state. It is not completely understood, among other details, when ATP binding or hydrolysis by Hsp90 occurs, and how clients are released from these complexes.

The precise temporal progression through Hsp90’s functional cycle is a matter of ongoing research (Figure 4), complicated (amongst others) by Hsp90’s client-specific behaviour, leading to many different functional cycles proposed in the literature (Verba et al., 2016; Lee et al., 2021; Wen et al., 2023; Kirschke et al., 2014). We focus here on two of the best-studied systems, the Hsp70-Hsp90 cascade and the kinase cycle, which both progress through three main phases: (i) client loading/recruitment, leading to (ii) client processing and maturation, followed by (iii) client release. Both examples show cochaperone-dependent client loading, but we note that several clients bind Hsp90 without cochaperone involvement, e.g., the IDPs Tau (Karagöz et al., 2014) and α-synuclein (Daturpalli et al., 2013). Also, due to the generally transient low-affinity interactions, numerous intermediate complexes and their interconversion kinetics are yet to be revealed and quantified. Therefore, as direct real-time observations are still lacking, the depicted cycles represent early models from which neither a strict order of events nor their timing can be inferred.

The Hsp70-Hsp90 cascade (Figure 4a) was found to process the transcription factors GR (Noddings et al., 2021; Wang et al., 2021; Kirschke et al., 2014; Morán Luengo et al., 2018) and p53 (Dahiya et al., 2019), as well as Argonaute which regulates transcription through RNA silencing (Tsuboyama et al., 2018; Pare et al., 2009; Pare et al., 2013; Wang et al., 2013). Clients are prepared for Hsp90 interaction through active (partial) unfolding by Hsp70 via hydrophobic interactions (Rüdiger et al., 1997), a mechanism supported by findings of single-molecule optical tweezer experiments (Dahiya et al., 2022; Moessmer et al., 2022). Two recently resolved multipartite intermediate states, the loading (Wang et al., 2021) and the maturation complex (Noddings et al., 2021), provide much awaited molecular-level structural details. The loading complex, Hsp70-Bridge-Client-Hsp90, contains a ‘bridging’ protein (e.g. Hop [Morán Luengo et al., 2018], NudC [Biebl et al., 2022], Tomm34 [Trcka et al., 2014]) that facilitates client transfer from Hsp70 to Hsp90. Interestingly, Hop was found not to be required in vivo (Bhattacharya et al., 2020) which may reflect redundancy among bridging proteins. Another bridging protein, NudC was found to directly recruit Hsp40-bound clients to Hsp90 (Biebl et al., 2022). Following recruitment, the partially unfolded client is clamped within the closed Hsp90 dimer lumen, and whether this is a directional or reversible step still needs to be determined by time-resolved techniques. Next, the Hsp90-client-p23 maturation complex (Noddings et al., 2021) is formed after dissociation of the bridging protein and Hsp70, where dissociation involves either nucleotide exchange or Hsp90 ATPase activity (Kirschke et al., 2014). p23 binds and stabilises Hsp90’s closed state (Richter et al., 2004) during GR processing, while it is not required for luciferase processing (Morán Luengo et al., 2018). Interestingly, an smFRET study reported directionality of an Hsp90/p23 system conferred by ATP binding and hydrolysis even in the absence of a client protein (Ratzke et al., 2014), implying that cochaperones can steer the Hsp90 cycle. Additionally, p23 binding was found to relieve a Hop-inhibited Hsp70-Hsp40-Hop-GRLBD-Hsp90 complex and maximise GR hormone binding recovery beyond the levels achieved by the binding of either Hop or p23 alone (Dahiya et al., 2022), further indicating cochaperone-endowed directionality in the Hsp70-Hsp90 cascade. Other cochaperones may further modulate client-specific regulation, as was shown for immunophilins in the context of GR (Lee et al., 2021; Noddings et al., 2023). Further, cochaperones themselves can be regulated by PTMs (e.g. Hop phosphorylation at residue Y354 reducing its binding to Hsp70/90 [Castelli et al., 2023]). The position of GR captured in the Hsp90 lumen shifts by several amino acids between the 3D-resolved loading and the maturation states, implying a sliding or ratcheting movement (Noddings et al., 2021). Although not all molecular details are known yet (e.g. on client release), the Hsp70-Hsp90 cascade appears to enable client activation through assisted folding (Kirschke et al., 2014; Morán Luengo et al., 2018).

Another functional cycle has been described for kinase-specific processing (Verba et al., 2016; Taipale et al., 2012; Figure 4b). Here, Cdc37 plays the client-recruiting role, where the loading complex consists of Cdc37-Client-Hsp90. Cdc37 stabilises a partially unfolded kinase state (Keramisanou et al., 2022; Keramisanou et al., 2016), in which the kinase N and C domains are separated (Verba et al., 2016), and thereby primes the kinase client for subsequent interaction with residues in the Hsp90 dimer lumen (Verba and Agard, 2017). Cdc37 can bind Hsp90 in its open state, inhibiting Hsp90’s ATPase through interaction with its ATP lid (Roe et al., 2004). In this loading complex, phosphorylation of serine 13 on Cdc37 (Cdc37-pSer13) by casein kinase 2 (Miyata and Nishida, 2004) is critical for kinase activation by Hsp90 (Miyata and Nishida, 2004; Shao et al., 2003), and stabilises the kinase-bound conformation (Verba et al., 2016). The maturation complex is obtained by binding of protein phosphatase 5 (Pp5), whereafter Pp5 dephosphorylates the kinase clients (Oberoi et al., 2022) as well as Cdc37-pSer13 (Vaughan et al., 2008; Oberoi et al., 2016). Steric hindrance in a cryo-EM structure of Hsp90-Cdc37-CRaf-PP5 suggests kinase dissociation prior to Cdc37 dephosphorylation and dissociation (Jaime-Garza et al., 2023). As Cdc37’s pSer13 residue is buried in the closed, ATP-bound Hsp90 dimer (Oberoi et al., 2022), the Pp5 dephosphorylation of Cdc37 likely happens upon Hsp90 transitioning from a compact ATP-bound state to a post-hydrolysis state, which is more flexible than the former (Graf et al., 2009). The Hsp90-Cdc37-kinase system has been described to process clients by ‘factory resetting’ of phosphorylations (Oberoi et al., 2022), as well as physical stabilisation or shielding of clients (Verba and Agard, 2017). Next to client PTMs, Hsp90 and Cdc37 are understood to undergo a series of modifications during the general kinase client cycle, which are suggested to provide directionality to the functional cycle (Xu et al., 2012). Another possible route of conferral of directionality, in addition to PTMs, is by cochaperones and ATP binding or hydrolysis, as revealed for the Hsp90-Cdc37-kinase cycle by smFRET, evaluated through direct observation of Hps90 open/closed dynamics (Ratzke et al., 2014; Vollmar et al., 2024). Most notably this was found for a complete reaction mixture, which included Hsp90, ATP, Cdc37, Sba1 (p23), Aha1, as well as the client kinase Ste11.

The timing of functional cycles is known to be crucial for biomolecular systems and their precise regulation. Prominent examples include: the pausing mechanism of RNA polymerase (Lerner et al., 2016; Janissen et al., 2022), ribosome rotation, stalling, and frameshifting during translation (Tsai et al., 2016), processive protein threading by proteases (Cordova et al., 2014), etc. For Hsp90, this level of mechanistic understanding has not yet been reached. Up to now, only some of the state transitions of the functional cycle could be observed experimentally, leaving a range of questions unanswered: When and for what is ATP binding required? When does ATP hydrolysis take place? Is ATP binding or hydrolysis or rather cochaperone phosphorylation dominant in providing directionality – and hence processivity – to a given Hsp90 cycle? In cellulo, do cochaperones interact with and dissociate from Hsp90 in a given sequence or randomly? What determines which client processing mechanism is employed? What is the timing of specific state transitions during client processing? Clearly, the time-resolved direct observation of Hsp90’s functional cycles would hold tremendous information about cycle timing, off-target waiting states, the client-specific differences, and the energetic driving forces of Hsp90’s functional cycle.

## Single-molecule observations complement the mechanistic understanding of Hsp90

Single-molecule methods have provided unique insight into Hsp90, and their abilities keep growing. By providing direct observations of individual proteins and protein complexes, they complement ensemble techniques (such as bulk enzymatic/activity assays, nuclear magnetic resonance, mass spectrometry, X-ray crystallography, etc.). Amongst others, single-molecule methods can uncover conformational heterogeneity normally hidden in ensemble averages, reveal reversible processes, elucidate the multiple reaction rates underlying the rate-limiting one, and quantitate the energies that drive molecular mechanisms. Amongst others, smFRET (Lerner et al., 2018) and force spectroscopies (Bustamante et al., 2021) are popular techniques that provide time-resolved access to protein functional determinants, such as intra- and inter-molecular dynamics (Lerner et al., 2018), molecular forces (Bustamante et al., 2021), enzymatic reactions (Lu et al., 1998), and movement (Yildiz et al., 2004) at single-molecule resolution.

Specifically, using smFRET, it was revealed that prokaryotic and eukaryotic Hsp90 are distinct regarding their conformational rearrangements, which are strongly ATP hydrolysis dependent in the prokaryotic (Ratzke et al., 2012b) but not eukaryotic (Mickler et al., 2009; Ratzke et al., 2012a) homologue. smFRET also showed that human Hsp90 exhibits greater structural flexibility on the nanosecond timescale compared to yeast Hsp90, which may explain why hHsp90 is regulated by a more diverse set of cochaperones than yeast Hsp90 (Riedl et al., 2024). Next, the conferral of directionality by cochaperones and ATP binding or hydrolysis (Ratzke et al., 2014; Vollmar et al., 2024) was uncovered by smFRET, and also how Hsp90’s conformational kinetics are regulated by environmental conditions (e.g. the presence of point mutations, crowding agents, or cochaperones [Schmid and Hugel, 2020]). In addition, it could be shown that Hsp90 inhibitors have little effect on Hsp90’s conformational dynamics (Schmid et al., 2018). Furthermore, smFRET revealed that the human Hsp90 paralog found in the endoplasmic reticulum, Grp94, exhibits two closed states, which was explained to arise from sequential ATP hydrolysis by the Hsp90 protomers (Huang et al., 2019). Also, smFRET elucidated that the yeast Hsp90 charged linker reduces the probability of the N-terminal dimerisation conformation of the Hsp90 dimer as compared to Hsp90 mutants lacking charged residues in the linker (Jahn et al., 2014). Moreover, smFRET showed that the p53 DBD exhibits considerable dynamics in the presence of all required chaperoning components (i.e. Hsp70, Hsp40, ATP, HOP, Bag1, and Hsp90), suggesting that p53 conformation is constantly remodelled by Hsp70/Hsp90 (Dahiya et al., 2019). In an integrative study, building on structural information, more than 100 FRET pairs on the Hsp90 dimer were used to disentangle local and global dynamics of the Hsp90 dimer, and it was found that the model client Δ131Δ only affects open state dynamics and does not affect open-closed interconversion dynamics (Hellenkamp et al., 2017). Another study connecting smFRET and molecular dynamics observations suggests that ATP hydrolysis leads to a conformational state in which the client binding site in the Hsp90 dimer lumen becomes constricted (Wolf et al., 2021). Another fluorescence-based technique, single-molecule photo-induced electron transfer fluorescence correlation spectroscopy (PET-FCS), revealed sub-millisecond ATP lid opening (release) by yHsp90 that was two- to threefold increased by Aha1 (Schulze et al., 2016). Using PET-FCS it was also found that three conformational motions in Hsp90 happen in concert, i.e., within ~2 s for NM closure and ATP lid closure, NM closure and NTD β-strand exchange, as well as ATP lid closure and NTD β-strand exchange (Schubert et al., 2021). Complementary to fluorescence, single-molecule force spectroscopy studies using optical tweezers have revealed the mechanism by which Hsp90 (itself) folds (Jahn et al., 2016), and elucidated the functional role of its flexible charged linker region (Jahn et al., 2014). Similar experiments have been used to perform a detailed comparison of Hsp90 orthologs, where large differences in the flexibility of the charged linker regions were identified (Jahn, 2018). Also, Hsp90 isoforms were investigated by force spectroscopy, where despite a very high sequence similarity, differences in stability, refolding capacity, and their conformational cycles were observed (Girstmair et al., 2019). Lastly, optical tweezer experiments revealed details of the ATP dependence of Hsp90’s dimerisation dynamics (Tych et al., 2018), of Hsp70-mediated client unfolding (Dahiya et al., 2022), and the local compaction of the model client luciferase in an ATP-dependent manner (Mashaghi et al., 2022). Altogether, by building on a large body of existing biochemical work, single-molecule techniques offer valuable complementary experiments that provided otherwise inaccessible insights through direct observations of single proteins at work.

Looking ahead, the future is bright for new approaches applied to Hsp90 – in part due to recent technological advances. For example, Hsp90’s weak interactions with clients and cochaperones posed a major challenge of single-molecule experiments in the past. This regime can now be studied using nanophotonic approaches such as zero-mode waveguides (ZMW) – which overcome previous detection limitations for single-molecule fluorescence spectroscopy (Tsai et al., 2016; Eid et al., 2009). Alternatively, tethered constructs, where the local concentration of a cochaperone, for example, is enhanced by connecting it to Hsp90 through an unstructured amino acid linker, are being used for both fluorescence spectroscopy and force-based measurements (Mondol et al., 2023). In fact, many of the urgent questions discussed herein could be clarified with the help of single-molecule experiments, and, in Box 1, we provide a non-exhaustive list with specific examples.

## Summary

In summary, the central chaperone Hsp90 is a peculiar ATPase differing drastically from other well-known ATPases, and it has remained surprisingly enigmatic. We highlighted three fundamental unanswered questions on Hsp90. *First*, what is the purpose of Hsp90’s ATPase activity? It has been revealed that Hsp90 can show widespread allostery in its hydrolysis mechanism. Additionally, Hsp90’s ATP hydrolysis was proposed to induce dissociation of interaction partners, but in cellulo, it was found that nucleotide exchange (not hydrolysis) was sufficient for several Hsp90 chaperoning functions. *Second*, what are the molecular actions by which the Hsp90 system processes its clients? First molecular-level insights from cryo-EM structures provided static evidence for dynamic rearrangements, such as sliding, stretching, and holding – which represent valuable hypotheses to test with time-resolved observations, e.g., using single-molecule techniques. *Third,* what are the kinetics of Hsp90’s functional cycle, or rather its multiple client-specific cycles? That is, what is the timing, order, and interdependence of events that describe this multi-component system? With first molecular structures elucidating parts of the functional cycle plus ample biochemical results on the interaction partners involved, it is now possible to set up experiments to directly observe these functional cycles by following a single multipartite complex over time.

Altogether, mechanistic insight on the multifaceted chaperone function of Hsp90 is urgently needed to inform and accelerate biomedical and pharmaceutical advances, targeting several conditions ranging from cancer to neurodegenerative diseases and more. Compared to other intensely studied protein systems, the understanding of the Hsp90 chaperone lags behind, because its transient multipartite complexes have been challenging to study in molecular detail. Nevertheless, a large body of biochemical knowledge has accumulated over the years, 3D structural information on the transient Hsp90 complexes is growing, and recent technological advances enable now direct observation of Hsp90 at work. Equipped with these, the time is better than ever to address and uncover the remaining mechanistic known unknowns of the Hsp90 chaperone.
